# Gut Microbiome Signatures Associated with Osteoporotic Vertebral Compression Fracture

**DOI:** 10.3390/ijms27198468

**Published:** 2026-09-23

**Authors:** Sujin Jo, Hoonhee Seo, Md Sarower Hossen Shuvo, Izaz Ahmed, Sukyung Kim, Je Hoon Jeong, Ho-Yeon Song

**Affiliations:** 1 Department of Microbiology and Immunology, School of Medicine, Soonchunhyang University, Cheonan-si 31151, Chungnam, Republic of Korea; sujiny0206@gmail.com (S.J.); iahmedhimel001@gmail.com (I.A.); 2 K-Microbiome Institute, 813-26 Yisunsin-daero, Asan 31462, Chungnam, Republic of Korea; eehh2015@hanmail.net (H.S.); kmshkj@naver.com (S.K.); 3 Department of Neurosurgery, Soonchunhyang University Bucheon Hospital, 170 Jomaru-ro, Bucheon 14584, Gyeonggi-do, Republic of Korea

**Keywords:** osteoporotic vertebral compression fracture, gut microbiome, osteoporosis, bone mineral density, short-chain fatty acids

## Abstract

Osteoporotic vertebral compression fracture (OVCF) is a clinically important condition associated with pain, disability, and impaired quality of life. Although osteoporosis is commonly diagnosed based on bone mineral density (BMD), fracture occurrence cannot be fully explained by BMD alone, suggesting that additional systemic factors may contribute to fracture susceptibility. The gut microbiome has recently emerged as a potential regulator of bone metabolism; however, its association with OVCF remains insufficiently characterized. In this study, fecal microbiome profiling using 16S rRNA gene sequencing was performed in 29 healthy controls, 11 patients with osteoporosis, and 25 patients with OVCF. Compared with the healthy control and osteoporosis groups, the OVCF group exhibited more distinct gut microbial compositional differences and reduced microbial diversity. Differential abundance analyses identified an increased relative abundance of *Alistipes* in the OVCF group and a higher relative abundance of *Prevotella* in healthy controls, with these taxa showing negative and positive correlations with BMD, respectively. Functional profiling further suggested differences in microbial metabolic pathways associated with OVCF, including increased mucin-derived carbohydrate degradation and reduced polyamine metabolism pathways. Consistently, fecal short-chain fatty acid concentrations were significantly lower in the OVCF group, accompanied by a reduced abundance of short-chain fatty acid-producing taxa and alterations in related microbial pathways. Overall, these findings suggest that gut microbial and metabolic alterations are associated with OVCF status rather than osteoporosis alone. The identified microbial signatures provide preliminary evidence of OVCF-associated microbial alterations; however, further validation in independent cohorts and longitudinal studies is required before their diagnostic or predictive value can be established.

## 1. Introduction

Osteoporosis is a systemic skeletal disorder characterized by reduced bone mass and deterioration of bone microarchitecture, leading to increased bone fragility and a higher risk of fracture [1]. The clinical significance of osteoporosis is primarily attributed to its association with osteoporotic fractures, which result in substantial morbidity, reduced quality of life, and increased healthcare burden. Among these, osteoporotic vertebral compression fracture (OVCF) is one of the most common complications and represents a major clinical manifestation of skeletal fragility [2]. Although BMD is a major diagnostic criterion for osteoporosis, a substantial proportion of patients with osteoporotic fractures do not have severe BMD reduction, and conversely, many patients with low BMD never experience fractures. This strongly indicates that non-BMD systemic factors contribute to skeletal fragility and fracture occurrence [3,4].

Bone homeostasis is regulated through a complex interplay of endocrine, immune, and metabolic pathways [5]. In this context, the gut microbiome has emerged as an important regulator of host physiology, influencing immune responses, nutrient metabolism, and the production of bioactive metabolites. Increasing evidence suggests that gut microbial communities may influence bone metabolism through modulation of systemic inflammation, intestinal barrier function, calcium absorption, and production of short-chain fatty acids (SCFAs) [6]. These observations have led to the concept of the gut–bone axis, highlighting the potential role of the gut microbiome in skeletal health. Consequently, alterations in the gut microbiome have been proposed as one of several biological factors that may be associated with bone fragility beyond BMD [3].

Previous studies have reported associations between gut microbiome composition and osteoporosis, including alterations in microbial diversity and specific taxa associated with BMD and metabolic status. Microbial metabolites, particularly SCFAs such as acetate, propionate, and butyrate, have also been implicated in bone metabolism through their roles in immune regulation and osteoclast activity [7]. However, previous microbiome studies of osteoporosis have yielded inconsistent or modest findings, suggesting that osteoporosis alone may not fully capture gut microbiome alterations associated with skeletal fragility. Furthermore, because osteoporosis is defined primarily by low BMD rather than fracture status, evaluating patients with established osteoporotic fractures may provide additional insight into microbiome alterations associated with clinically manifested skeletal fragility [8].

OVCF represents a clinically important complication of osteoporosis, bringing abrupt physiological stresses, pain, immobilization, and localized hyper-remodeling, in which skeletal fragility is accompanied by vertebral fracture. Therefore, comparing healthy controls (HC), patients with osteoporosis (OP), and patients with OVCF may help determine whether gut microbiome profiles differ among these clinically distinct groups. In the present study, we characterized the gut microbiome across HC, OP, and OVCF groups using 16S rRNA gene sequencing and explored microbial taxa, predicted functional pathways, and microbial metabolites associated with OVCF.

## 2. Results

### 2.1. Radiologic Assessment of Osteoporotic Vertebral Compression Fracture and Morphometric Analysis

Radiologic evaluation was performed to characterize OVCF and quantitatively assess fracture severity using standardized morphometric parameters (Figure 1). Dual-energy X-ray absorptiometry analysis demonstrated reduced BMD with osteoporotic-range T-scores, confirming the osteoporotic background associated with fracture susceptibility in enrolled patients (Figure 1A). Magnetic resonance imaging further revealed signal alterations and structural collapse of the affected vertebral body, confirming a vertebral compression fracture (Figure 1B). Consistent with these findings, bone scintigraphy showed increased radiotracer uptake localized to the fractured vertebral region, indicating active bone remodeling and metabolic activity at the injury site (Figure 1C). Lateral spine X-ray imaging additionally demonstrated vertebral body height loss and kyphotic deformity characteristic of OVCF in the sagittal plane (Figure 1D). To quantitatively evaluate fracture severity, anterior vertebral body heights of the superior vertebra (V_superior), injured vertebra (V_injured), and inferior vertebra (V_inferior) were measured using standardized morphometric methods (Figure 1E). Based on these measurements, compression ratio and wedge angle analyses were performed to assess the extent of vertebral deformity (Figure 1F). The mean wedge angle and compression ratio were 11.35° and 26.11%, respectively, indicating substantial vertebral compression in patients with OVCF.

### 2.2. Alterations in Gut Microbial Community Structure Based on Beta Diversity Analysis

Beta diversity analysis was performed to evaluate differences in gut microbial community structure among the HC, OP, and OVCF groups using principal coordinate analysis (PCoA) based on the Bray–Curtis, Jensen–Shannon, and Jaccard indices (Figure 2). PCoA plots showed distinct clustering of the OVCF group compared with the HC and OP groups across all beta-diversity metrics (Figure 2A). In contrast, the HC and OP groups largely overlapped within the ordination space, suggesting relatively similar microbial community compositions. Similar separation tendencies were consistently observed in analyses based on Bray–Curtis, Jensen–Shannon, and Jaccard indices. Pairwise PERMANOVA analysis further supported these findings (Figure 2B). No significant differences were observed between the HC and OP groups in the Bray–Curtis index (*p* = 0.324, FDR = 0.324), Jensen–Shannon divergence (*p* = 0.209, FDR = 0.209), or Jaccard index (*p* = 0.378, FDR = 0.378). In contrast, the OVCF group showed significant separation from both the HC and OP groups across all beta-diversity metrics. In the Bray–Curtis analysis, significant differences were identified for HC vs. OVCF (R^2^ = 0.079, *p* = 0.001, FDR = 0.002) and OP vs. OVCF (R^2^ = 0.113, *p* = 0.001, FDR = 0.002). Similar patterns were observed in the Jensen–Shannon divergence analysis, where HC vs. OVCF and OP vs. OVCF comparisons showed significant differences with higher explanatory power (R^2^ = 0.121 and 0.167, respectively; both FDR = 0.002). The Jaccard index analysis also demonstrated significant separation between the OVCF group and the other groups, whereas HC and OP remained non-significantly different. These findings indicate that gut microbial community structure was distinctly altered in the OVCF group compared with both the HC and OP groups.

### 2.3. Reduced Alpha Diversity in the OVCF-Associated Gut Microbiota

Alpha diversity analysis was performed to evaluate within-sample microbial richness and diversity among the HC, OP, and OVCF groups using multiple richness- and diversity-based indices (Figure 3). Richness-based indices, including Observed species, Chao1, and ACE, were analyzed to assess microbial richness within each group (Figure 3A). The HC and OP groups showed distributions that were relatively comparable across all richness indices, with substantial overlap in overall richness patterns. In contrast, the OVCF group demonstrated an overall reduction in microbial richness, with several samples distributed within lower richness ranges compared with HC and OP. Significant reductions were observed in both the Observed species and Chao1 indices in the OVCF group, indicating decreased microbial richness and estimated species abundance associated with vertebral fracture status. Although the ACE index did not reach statistical significance, a decreasing trend was similarly observed in the OVCF group relative to HC and OP. Diversity-based indices, including Shannon, Simpson, and Fisher indices, were subsequently analyzed to evaluate microbial diversity and evenness (Figure 3B). The Shannon and Simpson indices showed relatively similar distributions across the three groups, with no significant intergroup differences. However, the OVCF group showed greater variability and a tendency toward lower diversity in several samples. In contrast to the Shannon and Simpson indices, the Fisher index showed a significant reduction in the OVCF group compared with the HC and OP groups, suggesting decreased microbial diversity in OVCF. The distribution pattern further showed that HC and OP samples were generally clustered within relatively preserved diversity ranges, whereas OVCF samples were more frequently found in lower-diversity regions.

Reduced microbial richness and diversity were observed in the OVCF-associated gut microbiota.

**Figure 3:**
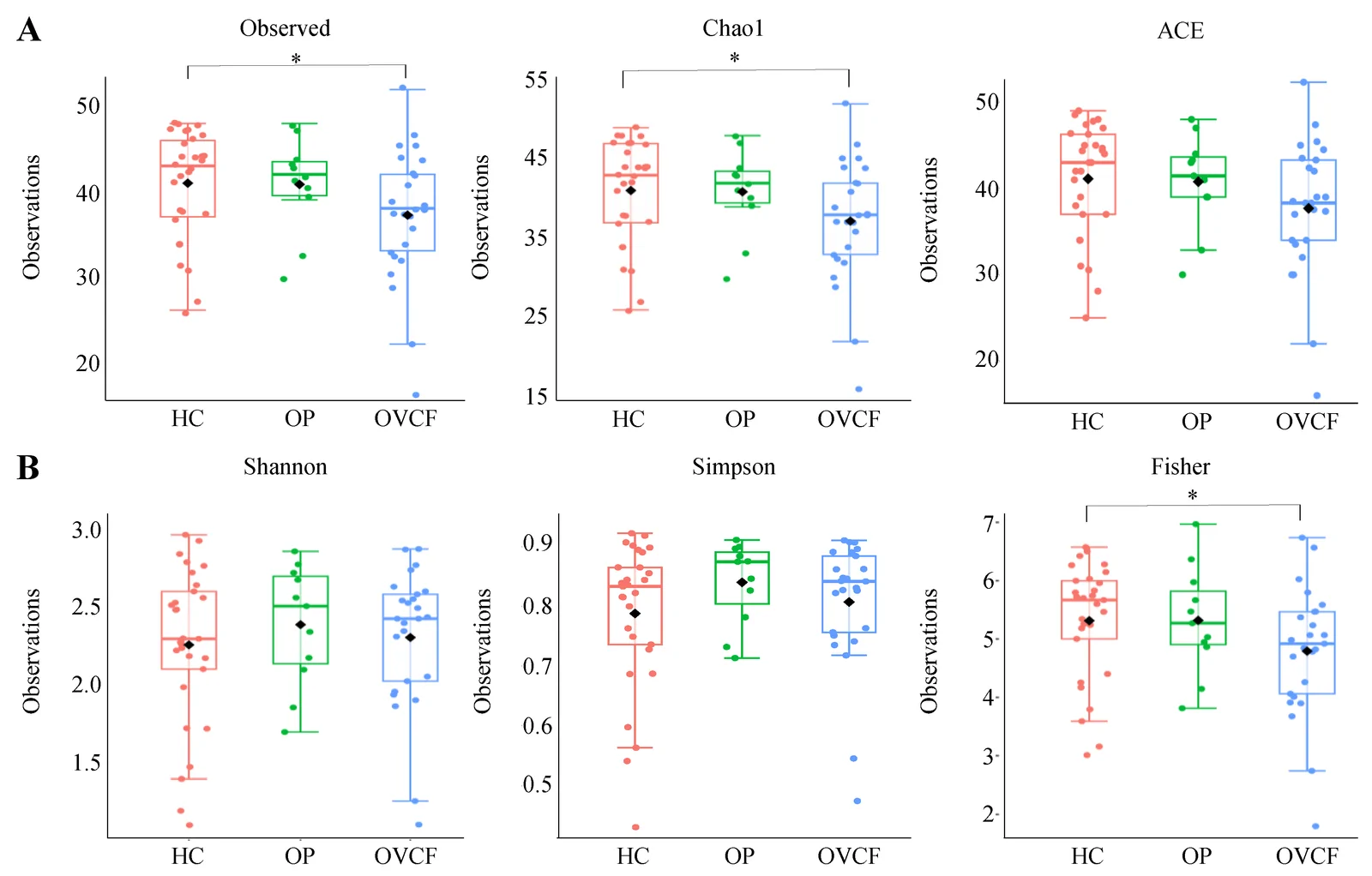
Alpha diversity differences in gut microbial communities across study groups. Alpha diversity analysis was performed to evaluate within-sample microbial richness and diversity among the three groups (healthy controls, HC; osteoporosis patients, OP; and osteoporotic vertebral compression fracture patients, OVCF). (**A**) Richness indices showed no significant differences between the HC and OP groups, whereas the OVCF group showed a decreasing trend, with significant reductions in Observed species and Chao1. (**B**) Diversity indices showed similar distributions between the HC and OP groups, whereas the OVCF group exhibited an overall decreasing trend, with a significant reduction only in the Fisher index; Shannon and Simpson indices were not significantly different. Statistical analysis was performed using nonparametric tests with FDR correction, and box plots show the median and interquartile range (IQR), with each point representing an individual sample. * indicates a statistically significant difference between groups (FDR-adjusted *p* < 0.05).

### 2.4. Taxonomic Alterations in Gut Microbial Composition Associated with OVCF

Taxonomic composition analysis was performed to compare relative abundances of gut microbial communities among the HC, OP, and OVCF groups across multiple taxonomic levels, including phylum, class, order, family, and genus (Figure 4). At the phylum level, the overall microbial composition was primarily dominated by Bacteroidetes and Firmicutes across all groups, without marked compositional shifts between groups. Similar distribution patterns were observed at the class and order levels, where *Bacteroidia* and *Clostridia*, as well as *Bacteroidales* and *Clostridiales*, remained the predominant taxa among all groups. Minor variations were observed in Gammaproteobacteria, Bacilli, *Enterobacteriales*, and *Bifidobacteriales*, although no clear global compositional separation was identified. At the family level, the three groups showed relatively similar abundance distributions, while moderate alterations were observed in several families, including *Ruminococcaceae*, *Lachnospiraceae*, and Enterobacteriaceae. More pronounced compositional alterations were identified at the genus level. The OVCF group showed increased relative abundances of Bacteroides and *Alistipes* compared with the HC group. In contrast, several genera associated with SCFA production and commensal gut microbial functions, including *Faecalibacterium*, *Prevotella*, *Subdoligranulum*, *Gemmiger*, *Ruminococcus*, and *Roseburia*, exhibited decreasing trends in the OVCF group. Additional compositional differences were also observed in *Dialister*, *Blautia*, *Veillonella*, and *Coprococcus* among the study groups. Overall, taxonomic compositional differences were relatively limited at higher taxonomic levels but became more distinct at the genus level in the OVCF-associated gut microbiota.

### 2.5. Differentially Abundant Microbial Taxa Identified by LEfSe Analysis

LEfSe analysis was performed to identify differentially abundant microbial taxa among the study groups based on linear discriminant analysis (LDA) scores with a threshold of ≥4 (Figure 5). Several taxa were enriched in the HC group compared with the OVCF group, including *Prevotella*, *Faecalibacterium*, *Dialister*, *Subdoligranulum*, *Romboutsia*, and *Clostridium sensu stricto 1* (Figure 5A). In contrast, *Bacteroides*, *Alistipes*, *Ruminococcaceae*, *Lachnoclostridium*, *UBA1819*, and *UCG_005* were enriched in the OVCF group. Among these taxa, *Bacteroides* and *Alistipes* showed the highest LDA scores in OVCF, whereas *Prevotella* and *Faecalibacterium* were strongly enriched in HC. In the comparison between OP and OVCF, the OP group showed enrichment of *Prevotella*, *Ruminococcus*, *Dialister*, *Romboutsia*, *Subdoligranulum*, *CAG_352*, *Escherichia-Shigella*, and *Faecalibacterium* (Figure 5B). Conversely, *Bacteroides*, *Alistipes*, *Parabacteroides*, *Lachnoclostridium*, *Blautia*, *Clostridia vadinBB60 group*, *UCG_005*, and *UBA1819* were enriched in the OVCF group. Similar to the HC comparison, *Bacteroides* and *Alistipes* were among the most prominently enriched taxa in OVCF. These results demonstrate distinct microbial signatures associated with OVCF, characterized by enrichment of Bacteroides- and Alistipes-associated microbial profiles and depletion of several commensal and SCFA-associated genera.

### 2.6. Differential Abundance Analysis of Gut Microbial Taxa Using edgeR

Differential abundance analysis was performed using edgeR to identify significantly altered microbial taxa among the HC, OP, and OVCF groups and to statistically validate microbial biomarkers identified by LEfSe analysis (Figure 6). Comparison between the HC and OVCF groups revealed multiple taxa that were significantly altered based on log2 fold change analysis (Figure 6A). Several taxa showed negative log2 fold change values, indicating enrichment in the HC group, whereas positive values represented taxa enriched in the OVCF group. Among the taxa overlapping with LEfSe-derived biomarkers, *Prevotella* and *Dialister* were consistently enriched in HC, while *Alistipes* showed marked enrichment in OVCF. In addition to these representative biomarkers, multiple taxa showed directional abundance shifts between the two groups, indicating broad alterations in microbial composition associated with vertebral compression fracture status. The distribution pattern further demonstrated that HC-associated taxa were largely clustered within the negative fold change range. In contrast, OVCF-associated taxa were predominantly distributed in the positive fold change region. A similar trend was observed in the comparison between OP and OVCF (Figure 6B). Several microbial taxa exhibited significant differential abundance between the two groups, with distinct directional separation according to log2 fold change values. Among the overlapping taxa identified by both edgeR and LEfSe analyses, *Dialister* was enriched in the OP group, whereas *Alistipes* remained consistently enriched in the OVCF group. Notably, OVCF-associated taxa generally exhibited higher positive fold change values compared with OP-associated taxa, suggesting more pronounced microbial compositional alterations in the fracture-associated microbiota. The clustering pattern also indicated that taxa enriched in OVCF were distributed separately from OP-associated taxa across the fold change spectrum. Overall, edgeR-based differential abundance analysis consistently supported the microbial patterns identified by LEfSe, demonstrating enrichment of *Alistipes*-associated microbial signatures in OVCF and depletion of several commensal taxa, including *Prevotella* and *Dialister*.

### 2.7. Target-Specific qPCR Validation

Target-specific qPCR was performed for *Alistipes* and *Prevotella* to further assess the sequencing-based findings (Figure S1). *Alistipes* showed significantly lower Cq values in the OVCF group than in the HC group (FDR-adjusted *p* = 0.033), indicating greater detection of *Alistipes* DNA in OVCF samples and supporting the direction observed in the 16S rRNA gene sequencing analysis. In contrast, the difference in *Prevotella* between HC and OVCF did not remain statistically significant after FDR correction (FDR-adjusted *p* = 0.074), indicating that the sequencing-associated pattern for *Prevotella* was not reproduced by target-specific qPCR.

### 2.8. Correlation Between Bone Mineral Density and Gut Microbial Biomarkers

Spearman correlation analysis was performed to evaluate the association between bone mineral density (BMD) and the relative abundance of representative gut microbial biomarkers identified in taxonomic and differential abundance analyses (Figure 7). A significant negative correlation was observed between BMD and the relative abundance of *Alistipes*, indicating that increased abundance of *Alistipes* was associated with lower BMD values (ρ = −0.394, *p* = 0.001) (Figure 7A). Samples with higher *Alistipes* abundance were predominantly distributed within the low-BMD range, consistent with the previously identified enrichment pattern in the OVCF group. In contrast, *Prevotella* exhibited a positive correlation with BMD (ρ = 0.251, *p* = 0.048), suggesting that increased abundance of *Prevotella* was associated with relatively preserved bone mineral density (Figure 7B). Higher *Prevotella* abundance was more frequently observed in samples with comparatively higher BMD values, consistent with its enrichment tendency in the HC-associated microbial profile. *Subdoligranulum* showed a significant negative correlation with BMD (ρ = −0.340, *p* = 0.006) (Figure 7C). Although this genus demonstrated statistical significance in correlation analysis, its directional association was not fully consistent with the biomarker enrichment patterns identified in earlier taxonomic analyses. In contrast, *Dialister* did not show a statistically significant correlation with BMD despite a weak positive association trend (ρ = 0.198, *p* = ns) (Figure 7D). The distribution pattern of *Dialister* abundance showed substantial variability across samples without a clear linear relationship with BMD values. Overall, these findings suggest that specific microbial biomarkers, particularly *Alistipes* and *Prevotella*, were significantly associated with bone mineral density and may reflect alterations in the microbiome linked to osteoporosis-related vertebral fracture status.

### 2.9. Functional Alterations in Gut Microbial Metabolic Pathways Associated with OVCF

Functional metabolic pathway analysis was performed using MetaCyc-based prediction profiling combined with principal component analysis (PCA) and random forest classification to evaluate functional differences in gut microbial communities among the HC, OP, and OVCF groups (Figure 8). PCA based on predicted MetaCyc pathway profiles demonstrated partial overlap between the HC and OP groups, whereas the OVCF group exhibited a relatively distinct distribution pattern within the ordination space (Figure 8A). The OVCF samples were more densely clustered within a separate region than the HC and OP samples, indicating alterations in predicted microbial metabolic functions associated with vertebral fracture status. In contrast, HC and OP showed broader overlap and less distinct functional separation. Random forest analysis was subsequently performed to identify key functional pathways contributing to discrimination between groups (Figure 8B). Pathways associated with polyamine biosynthesis, including the superpathway of polyamine biosynthesis (POLYAMSYN-PWY) and arginine/polyamine biosynthesis (ARG+POLYAMINE-SYN), showed the highest mean decrease accuracy values. In addition, pathways related to carbohydrate and glucuronate metabolism, including fucose degradation and catabolism pathways (FUCCAT-PWY and FUC-RHAMCAT-PWY), glucuronate catabolism (GLUCUROCAT-PWY), and galactose-associated metabolism (GALACT-), were identified as important contributors to group discrimination. Several additional pathways related to microbial carbohydrate utilization and intermediary metabolic processing, including PWY490-3, PWY-5005, PWY0-166, and PWY-7332, also demonstrated differential importance among the study groups. The pathway distribution patterns further demonstrated reduced representation of several HC-associated metabolic pathways in the OVCF group, whereas distinct metabolic pathway signatures were relatively enriched in the OVCF group. Overall, functional pathway profiling revealed measurable differences in predicted microbial metabolic activities among the study groups.

### 2.10. Altered Fecal Short-Chain Fatty Acid Profiles and Their Association with Bone Mineral Density

Fecal short-chain fatty acid (SCFA) concentrations were quantified to evaluate metabolic differences among the HC, OP, and OVCF groups, and correlation analysis was subsequently performed to assess their association with bone mineral density (BMD) (Figure 9). Comparison of fecal SCFA concentrations demonstrated overall reductions in acetate, butyrate, and propionate levels in the OVCF group compared with the HC and OP groups (Figure 9A). Acetate concentrations showed a marked decreasing trend in OVCF relative to HC and OP. Similar reduction patterns were observed for butyrate and propionate, with OVCF samples consistently exhibiting lower fecal concentrations than the other groups. In contrast, valerate levels did not show a comparable decreasing pattern and remained relatively preserved across groups. Spearman correlation analysis further demonstrated significant positive associations between several SCFAs and BMD values (Figure 9B). Acetate showed the strongest positive correlation with BMD (ρ = 0.510, *p* = 0.004), indicating that higher acetate concentrations were associated with relatively preserved bone mineral density. Butyrate also demonstrated a significant positive correlation with BMD (ρ = 0.404, *p* = 0.027). Propionate showed a positive association with BMD (ρ = 0.354), although the difference did not reach statistical significance. In contrast, valerate showed no significant correlation with BMD and demonstrated a weak negative association trend (ρ = −0.165). The distribution patterns also showed that samples from the OVCF group were generally clustered within lower ranges of acetate, butyrate, and propionate concentrations, along with lower BMD values. In contrast, HC samples were more frequently associated with higher SCFA concentrations and relatively preserved BMD. Overall, fecal acetate, butyrate, and propionate levels were reduced in the OVCF group and were positively associated with bone mineral density.

## 3. Discussion

In the present study, we investigated differences in the gut microbiome across HC, OP, and OVCF groups. Overall, differences between HC and OP were relatively modest across taxonomic, diversity, and predicted functional analyses, whereas the OVCF group exhibited more distinct microbial profiles [9,10]. These differences were observed at the compositional level and were accompanied by differences in predicted metabolic pathways and fecal SCFA profiles. Furthermore, several microbial taxa and metabolites were significantly associated with BMD. Collectively, these findings identify gut microbial and metabolic features associated with OVCF and provide a basis for further investigation of the gut–bone axis in patients with osteoporotic fractures [11].

Recent advances in gut–bone axis research have also highlighted the potential therapeutic implications of microbiota-derived extracellular vesicles. Experimental evidence suggests that gut microbiota-derived bacterial extracellular vesicles can reach bone and influence bone remodeling in osteoporotic models [12]. In addition, engineered probiotic-derived bacterial extracellular vesicles are being explored as potential therapeutic platforms for osteoporosis [13,14]. These advances further underscore the translational significance of identifying gut microbial alterations associated with osteoporotic fractures.

Radiologic and morphometric analyses confirmed the presence and severity of vertebral fractures in the OVCF group. In addition to reduced BMD, MRI and lateral X-ray findings demonstrated vertebral compression deformities, while bone scintigraphy showed increased metabolic activity localized to affected vertebrae [15,16]. These findings provided radiological confirmation of OVCF and allowed the microbiome profiles of patients with clinically established vertebral fractures to be compared with those of individuals with osteoporosis without vertebral fracture and healthy controls. However, because fracture occurrence is influenced by multiple clinical and biological factors beyond BMD, the observed microbial differences should be interpreted as associations with OVCF status rather than as direct indicators of fracture causation. In addition, clinical factors accompanying OVCF, including fracture-related hospitalization, reduced mobility, analgesic use, and other changes associated with acute fracture, may also influence gut microbiome composition. Because these factors were not systematically assessed or controlled in the present study, their potential contribution to the observed microbial differences cannot be excluded.

One of the major findings of this study was the relatively limited separation between HC and OP in beta-diversity analyses [9]. In contrast, the OVCF group exhibited greater compositional differences from the other groups. Previous microbiome studies investigating osteoporosis have reported heterogeneous findings regarding microbial diversity and composition [17]. Several studies have reported reduced diversity or altered microbial composition in patients with osteoporosis, whereas others have observed relatively limited differences between healthy controls and individuals with low BMD. The substantial overlap between HC and OP observed in the present study is consistent with the variability reported in previous studies and suggests that osteoporosis defined primarily by BMD may not necessarily be accompanied by pronounced microbiome-wide differences in all populations. In contrast, the distinct profiles observed in the OVCF group indicate that patients with established osteoporotic vertebral fractures may have gut microbial characteristics that differ from those of individuals without fracture [18]. These differences may be associated with various clinical and systemic factors accompanying OVCF, including inflammatory, metabolic, and physiological changes [19]. Nevertheless, because of the cross-sectional design of this study, it cannot be determined whether these factors contributed to the observed microbial differences or resulted from clinical conditions associated with fracture. The consistent separation observed across Bray–Curtis, Jensen–Shannon divergence, and Jaccard analyses supports the presence of community-level differences in the OVCF group.

Alpha diversity analysis similarly demonstrated lower microbial richness in the OVCF group. Significant reductions in Observed species, Chao1, and Fisher indices indicated lower microbial richness in OVCF compared with the other study groups [20]. Previous osteoporosis-associated microbiome studies have reported heterogeneous findings regarding microbial diversity. While some studies have observed reduced microbial richness or diversity in patients with osteoporosis, others have reported limited or inconsistent differences across diversity indices and study populations [20]. Such variability may reflect differences in cohort characteristics, sample size, dietary and medication-related factors, and sequencing or analytical approaches. In the present study, reductions in richness-related indices were more apparent in the OVCF group than in the OP group. However, Shannon and Simpson indices did not differ significantly, suggesting that microbial evenness was relatively preserved despite differences in richness. These findings complement the beta-diversity results by demonstrating reduced microbial richness in the OVCF group.

Taxonomic composition analysis further demonstrated that differences associated with OVCF were relatively limited at higher taxonomic levels but became more apparent at the genus level. The overall dominance of Bacteroidetes and Firmicutes was maintained across all groups, indicating the absence of broad phylum-level restructuring. Instead, differences were observed among specific genera. In particular, *Bacteroides* and *Alistipes* showed higher relative abundance in the OVCF group, whereas several SCFA-associated genera, including *Faecalibacterium*, *Prevotella*, *Subdoligranulum*, *Ruminococcus*, and *Roseburia*, showed lower relative abundance. Previous studies have reported associations between reduced abundance of SCFA-producing genera and impaired bone metabolism or osteoporosis-related phenotypes [21]. Several genera that were less abundant in the OVCF group, including *Faecalibacterium*, *Roseburia*, and *Ruminococcus*, are recognized contributors to butyrate production and intestinal metabolic homeostasis [22]. The lower abundance of these genera may therefore be consistent with the differences in fecal SCFA profiles observed in the OVCF group. However, these associations do not establish a direct functional relationship between specific microbial taxa and fracture status.

LEfSe analysis further identified differentially abundant microbial taxa among the study groups. Several genera with higher relative abundance in HC and OP, including *Prevotella*, *Faecalibacterium*, *Dialister*, and *Subdoligranulum*, have been associated with carbohydrate fermentation and SCFA production [20]. In contrast, Bacteroides, *Alistipes*, and *Lachnoclostridium* showed higher relative abundance in the OVCF group. Among these taxa, *Alistipes* has previously been associated with inflammatory and metabolic conditions, whereas *Prevotella* and *Faecalibacterium* have been linked to carbohydrate fermentation and SCFA production [23]. These findings further support the distinct microbial profile observed in the OVCF group, although their biological significance requires further investigation.

EdgeR analysis showed directional abundance differences that were generally consistent with taxa identified by LEfSe. *Alistipes* showed higher relative abundance in the OVCF group, whereas *Prevotella* and *Dialister* were more strongly associated with the HC and OP groups. The concordance between the two analytical approaches supports the reproducibility of the observed differential abundance patterns within the present dataset [24]. Therefore, taxa identified by both LEfSe and edgeR were considered more robust findings than those detected by only one method. However, because both analyses were performed using the same cohort, validation in an independent population is required before these taxa can be considered robust OVCF-associated microbial markers.

Correlation analysis between selected microbial taxa and BMD demonstrated additional associations with bone-related clinical characteristics. *Alistipes* was negatively associated with BMD, whereas *Prevotella* showed a positive correlation with BMD [25,26]. These findings suggest that specific microbial taxa may be associated with both OVCF status and bone-related clinical parameters. However, these correlations should be interpreted cautiously, as they do not establish causal relationships and may be influenced by demographic and clinical factors. *Subdoligranulum*, for example, showed a significant negative association with BMD despite a directional pattern that was not fully consistent with the group-based taxonomic analyses. This finding further highlights the complexity of relationships between individual microbial taxa and host clinical characteristics.

Predicted functional pathway analysis indicated that the compositional differences observed in the OVCF group were accompanied by differences in predicted microbial metabolic potential. Similar to the beta-diversity results, HC and OP showed relatively overlapping predicted functional profiles, whereas the OVCF group exhibited greater pathway-level differences. In particular, pathways related to polyamine biosynthesis showed lower predicted representation in OVCF, whereas pathways involving fucose degradation, glucuronate catabolism, and mucin-derived carbohydrate metabolism showed differential representation. Polyamines contribute to cellular proliferation, intestinal epithelial integrity, immune regulation, and tissue repair, and previous studies have suggested potential links between polyamine metabolism and bone remodeling [27]. Altered representation of these pathways may therefore indicate differences in host–microbial metabolic interactions in patients with OVCF. Recent studies have further demonstrated that gut microbial alterations can be accompanied by changes in microbial metabolites, intestinal barrier function, and host inflammatory responses, highlighting the complex interplay between microbial communities and host physiology [28,29]. These findings provide a broader biological framework for interpreting the predicted functional and metabolic alterations observed in OVCF, although the underlying mechanisms remain to be experimentally validated. Similarly, differences in predicted mucin-derived carbohydrate metabolism may reflect altered microbial substrate utilization [30]. Because these functional pathways were inferred from 16S rRNA gene sequencing data using PICRUSt2, they represent predicted functional potential rather than direct measurements of microbial metabolic activity and should therefore be considered hypothesis-generating. Further validation using shotgun metagenomics, metabolomics, metatranscriptomics, or targeted functional assays is required [31].

The SCFA analysis provided additional evidence of metabolic differences among the study groups. Acetate, butyrate, and propionate concentrations were lower in the OVCF group, while acetate and butyrate showed positive associations with BMD. SCFAs are microbial metabolites involved in immune regulation, intestinal barrier maintenance, and host metabolic homeostasis, and previous studies have suggested potential roles in bone metabolism and osteoclast regulation [7,32]. In the present study, lower SCFA concentrations in the OVCF group were accompanied by lower relative abundance of several SCFA-associated genera, including *Faecalibacterium*, *Prevotella*, *Subdoligranulum*, and *Roseburia* [33]. Differences in predicted carbohydrate metabolism pathways were also observed. Taken together, these findings indicate coordinated differences in microbial composition, predicted metabolic potential, and fecal SCFA profiles in the OVCF group. Nevertheless, the cross-sectional nature of these observations does not allow determination of whether altered SCFA metabolism precedes OVCF or represents a secondary feature associated with differences in host characteristics or clinical status. In addition, dietary intake, including dietary fiber consumption, was not assessed in the present study and therefore could not be incorporated into the analysis. Because dietary fiber is an important determinant of microbial SCFA production, the observed differences in fecal SCFA concentrations may have been influenced by unmeasured dietary factors and should be interpreted with caution.

This study has several limitations. First, the sample size was relatively modest, particularly in the OP group (n = 11). The limited number of participants may have reduced the statistical power to detect subtle microbiome differences between HC and OP and may also have affected differential abundance analyses, potentially increasing the risk of false-positive or false-negative findings. Accordingly, the absence of significant beta-diversity differences between the HC and OP groups may partly reflect limited statistical power resulting from the relatively small OP sample size, rather than necessarily indicating the absence of biological differences between the HC and OP groups. Second, differences in demographic characteristics, including age, sex, and BMI distribution, among the study groups may have influenced gut microbiome composition and represent potential confounding factors. Detailed information regarding dietary intake, medication use, and other lifestyle and clinical factors that may affect the gut microbiome was also limited. Therefore, the observed microbial differences cannot be attributed specifically to OVCF independently of these potential confounders. Future studies using appropriately matched cohorts and multivariable analyses are required to evaluate the independent association between OVCF and gut microbiome characteristics. Third, because this was a cross-sectional study, causal or temporal relationships between gut microbiome alterations, SCFA profiles, and OVCF cannot be established. Fourth, functional pathway profiles were predicted from 16S rRNA gene sequencing data and therefore require validation using shotgun metagenomics, metatranscriptomics, or targeted functional assays. Fifth, although significant correlations were observed between selected microbial taxa, SCFAs, and BMD, these correlation analyses were exploratory in nature and were not adjusted for multiple testing. Therefore, the observed associations should be interpreted with caution and require validation in larger independent cohorts. Moreover, these correlations do not establish causal relationships and should not be interpreted as evidence that alterations in specific microbial taxa directly influence BMD or OVCF development. Finally, although fecal SCFA concentrations were measured, further studies integrating circulating metabolites, bone turnover markers, and mechanistic experimental models are needed to clarify the biological relevance of the observed gut microbial and metabolic differences in OVCF.

In conclusion, differences in gut microbiome profiles between HC and OP were relatively modest, whereas more distinct compositional, predicted functional, and metabolic differences were observed in the OVCF group [34]. These findings identify a set of gut microbial and metabolic characteristics associated with OVCF. Importantly, because this study did not include an independent validation cohort or diagnostic performance analyses, these findings should be considered preliminary and require further validation before their diagnostic or predictive value can be established. However, given the cross-sectional study design and potential demographic and clinical confounding factors, given the observational nature of the present study, the microbial patterns identified here cannot establish a direct role of the gut microbiome in fracture development or susceptibility. Their clinical and biological relevance should be further evaluated in appropriately matched populations using longitudinal designs and experimental approaches [35]. Figure 10 provides an overview of the principal gut microbial features observed in association with OVCF in this study.

## 4. Materials and Methods

### 4.1. Study Design, Patients, and Clinical Assessment

Participants were enrolled at Soonchunhyang University Bucheon Hospital between August 2021 and December 2023 in collaboration with the Probiotics Microbiome Convergence Center, Soonchunhyang University, Asan, South Korea. The study protocol was reviewed and approved by the Institutional Review Board (IRB No. 2025-04-018). A total of 65 participants were classified according to their clinical status into healthy control (HC), osteoporosis without vertebral fracture (OP), and osteoporotic vertebral compression fracture (OVCF) groups. For the OVCF group, fecal samples were specifically collected within 2–3 days after the occurrence of an acute vertebral fracture. Eligibility for this group required a single-level osteoporotic vertebral fracture without spinal cord compression, as confirmed by magnetic resonance imaging (MRI), and a bone mineral density (BMD) T-score below −2.5. Patients with neurological deficits, pathological fractures, unstable vertebral fractures involving the posterior spinal column, or a history of spinal procedures such as vertebroplasty were excluded. Clinical and radiological data were obtained from the Biobank of Soonchunhyang University Bucheon Hospital, a member of the Korea Biobank Network (2024ER050100). The compression ratio was defined as follows: first, calculating the ratio of the anterior height of the compromised vertebra (Vinjured) divided by the mean anterior height of the superiorly (Vsuperior) and inferiorly (Vinferior) adjacent vertebrae; then subtracting this ratio from 1 and multiplying by 100%. The kyphotic angle was defined as the angle subtended by the superior endplate of the vertebral body directly above and by the inferior endplate of the vertebral body directly below the fractured vertebra.

### 4.2. 16S rRNA Gene Sequencing and Microbiome Analysis

16S rRNA gene sequencing and bioinformatics analyses were performed as previously described [9]. Metagenomic DNA was extracted from fecal samples using the QIAamp DNA PowerFecal Pro DNA Kit (QIAGEN GmbH, Hilden, Germany) according to the manufacturer’s protocol. DNA concentration and purity were quantified using a Qubit 4 Fluorometer (Thermo Fisher Scientific, Waltham, MA, USA), and DNA integrity was assessed by agarose gel electrophoresis. Purified DNA samples were stored at −20 °C until further analysis. Extraction blanks were included as negative controls in each batch, and no detectable DNA was observed. Amplicon libraries targeting the V4 region of the bacterial 16S rRNA gene were prepared using the primer pair 515F (5′-GTGCCAGCMGCCGCGGTAA-3′) and 806R (5′-GGACTACHVGGGTWTCTAAT-3′) [36]. PCR amplification was performed using 2× KAPA HiFi HotStart ReadyMix (Kapa Biosystems (Pty) Ltd., Cape Town, South Africa) under optimized cycling conditions. The resulting amplicons were purified using AMPure XP beads (Beckman Coulter, Brea, CA, USA) to remove residual primers and small fragments, and purified libraries were quantified and pooled at equimolar concentrations prior to sequencing. Sequencing was performed on the Illumina iSeq 100 platform (Illumina, Inc., San Diego, CA, USA) according to the manufacturer’s standard protocols, generating paired-end reads for downstream analysis. Raw sequencing data were processed using the QIIME 2 pipeline [37]. A manifest file was generated in TSV format, and sequence reads were imported as SampleData[SequencesWithQuality] using the SingleEndFastqManifestPhred33V2 format. Quality control, denoising, and amplicon sequence variant (ASV) inference were performed using the DADA2 plugin with trim-left set to 0 and trunc-len set to 240 [38]. Taxonomic assignment was conducted using a Naive Bayes classifier trained on the SILVA 138 99% reference database. Low-abundance and low-prevalence features were filtered prior to downstream analyses to reduce the influence of sparse signals and sequencing noise [39]. Microbial diversity was assessed at both alpha and beta levels. Alpha diversity was evaluated using observed features, Chao1, ACE, Shannon, Simpson, and Fisher indices. For comparisons among the three groups, the Kruskal–Wallis test followed by Dunn’s multiple comparison test was used. For pairwise comparisons, the Mann–Whitney U test was applied where appropriate. *p*-values were adjusted using the false discovery rate (FDR) method when multiple comparisons were performed. Beta diversity was calculated using Bray–Curtis distance, Jensen–Shannon divergence, and Jaccard distance, and visualized by principal coordinates analysis (PCoA). Differences in community composition among groups were tested using permutational multivariate analysis of variance (PERMANOVA), with additional pairwise comparisons where appropriate [40,41,42]. No covariate adjustment was applied in the beta-diversity analysis. Because detailed information on dietary habits, medication use, and comorbidities was not consistently available, and this was an exploratory microbiome study, the results should be interpreted as unadjusted group comparisons. Taxonomic composition was summarized at multiple levels, including phylum, class, order, family, and genus. Differentially abundant taxa between groups were identified using linear discriminant analysis effect size (LEfSe) and edgeR with false discovery rate (FDR) correction [43,44]. LEfSe was used to identify taxa with discriminative effect sizes, whereas edgeR was used to statistically evaluate differential abundance with false discovery rate (FDR) correction. Taxa identified by both methods were considered more robust findings for subsequent analyses. Random forest analysis was additionally performed to identify key microbial taxa contributing to group discrimination, and feature importance was evaluated using mean decrease in accuracy. Functional profiles of the gut microbiome were predicted based on MetaCyc pathway annotations using the MicrobiomeAnalyst platform. Predicted pathway abundances were used for downstream analyses after filtering and normalization within the pipeline [45,46]. Principal component analysis (PCA) was performed to assess overall functional differences among groups, and random forests were used to identify pathways contributing to group discrimination [47]. Univariate comparisons were conducted as supplementary analyses. Multivariable analyses adjusting for age, sex, and body mass index (BMI) were additionally performed; however, no pathways remained statistically significant after adjustment.

### 4.3. Target-Specific Quantitative PCR (qPCR)

Target-specific quantitative PCR (qPCR) was performed to assess *Alistipes* and *Prevotella*, which were identified as key differentially abundant taxa in the 16S rRNA gene sequencing analysis. qPCR reactions were performed using taxon-specific primers, with each sample analyzed in technical triplicate. The mean Cq value of the technical replicates was used for statistical analysis. Lower Cq values indicate greater detection of the target bacterial DNA. Because a universal bacterial reference target was not included, the qPCR results were interpreted as target-specific Cq comparisons rather than normalized relative abundance measurements. Group differences were evaluated using the Kruskal–Wallis test followed by pairwise Mann–Whitney U tests, with *p*-values adjusted using the Benjamini–Hochberg false discovery rate (FDR) method.

### 4.4. Short-Chain Fatty Acid (SCFA) Analysis

Short-chain fatty acid (SCFA) analysis was performed according to our previous laboratory reports with minor modifications [48]. For SCFA quantification, 10 fecal samples were selected at random from each study group. Because the OP group consisted of only 11 participants, an equal number of samples (n = 10 per group) was analyzed to ensure balanced group comparisons. Briefly, fecal samples were subjected to methanol-based extraction and chemical derivatization prior to gas chromatography–tandem mass spectrometry (GC–MS/MS) analysis using a GC-2010 Plus system coupled to a TQ8040 triple quadrupole mass spectrometer (Shimadzu, Kyoto, Japan) equipped with a DB-5MS capillary column (30 m × 0.25 mm, 0.25 μm film thickness; Agilent Technologies, Santa Clara, CA, USA). For sample preparation, 20 mg of fecal sample was mixed with 0.1 M NaOH and methanol, incubated at −20 °C, and centrifuged to obtain the supernatant. The extracted samples were dried under vacuum and derivatized using methoxyamine hydrochloride and N-tert-butyldimethylsilyl-N-methyltrifluoroacetamide (MTBSTFA). The derivatized extracts were subsequently analyzed by GC–MS/MS under optimized chromatographic and mass spectrometric conditions. The GC oven temperature was programmed from 60 °C to 325 °C using a stepwise temperature gradient. Helium was used as the carrier gas, and analytes were detected in multiple reaction monitoring (MRM) mode with optimized precursor-to-product ion transitions for each SCFA. Acetate, propionate, butyrate, and valerate were quantified using external calibration curves generated from authentic SCFA standards subjected to identical derivatization procedures. To ensure analytical reproducibility and minimize carryover, blank samples and standard mixtures were included throughout the analytical sequence under standardized quality control conditions.

## 5. Conclusions

This study identified distinct gut microbial, predicted functional, and metabolic profiles in patients with OVCF compared with individuals with osteoporosis without vertebral fracture and healthy controls. The higher relative abundance of Alistipes, lower abundance of Prevotella and several SCFA-associated taxa, and reduced fecal SCFA concentrations characterized the OVCF group. Differences in predicted microbial pathways, including mucin-derived carbohydrate degradation and polyamine metabolism, further suggested alterations in microbial metabolic potential associated with OVCF.

Collectively, these findings identified gut microbial and metabolic features associated with OVCF but do not establish their causal involvement in fracture development. Because this study did not include an independent validation cohort or diagnostic performance analyses, these findings should be considered preliminary. Further studies using appropriately matched cohorts, longitudinal designs, independent validation cohorts, and mechanistic investigations are required to determine whether these microbial characteristics have clinical utility as biomarkers and to clarify their potential role in the gut–bone axis.

## Figures and Tables

**Figure 1:**
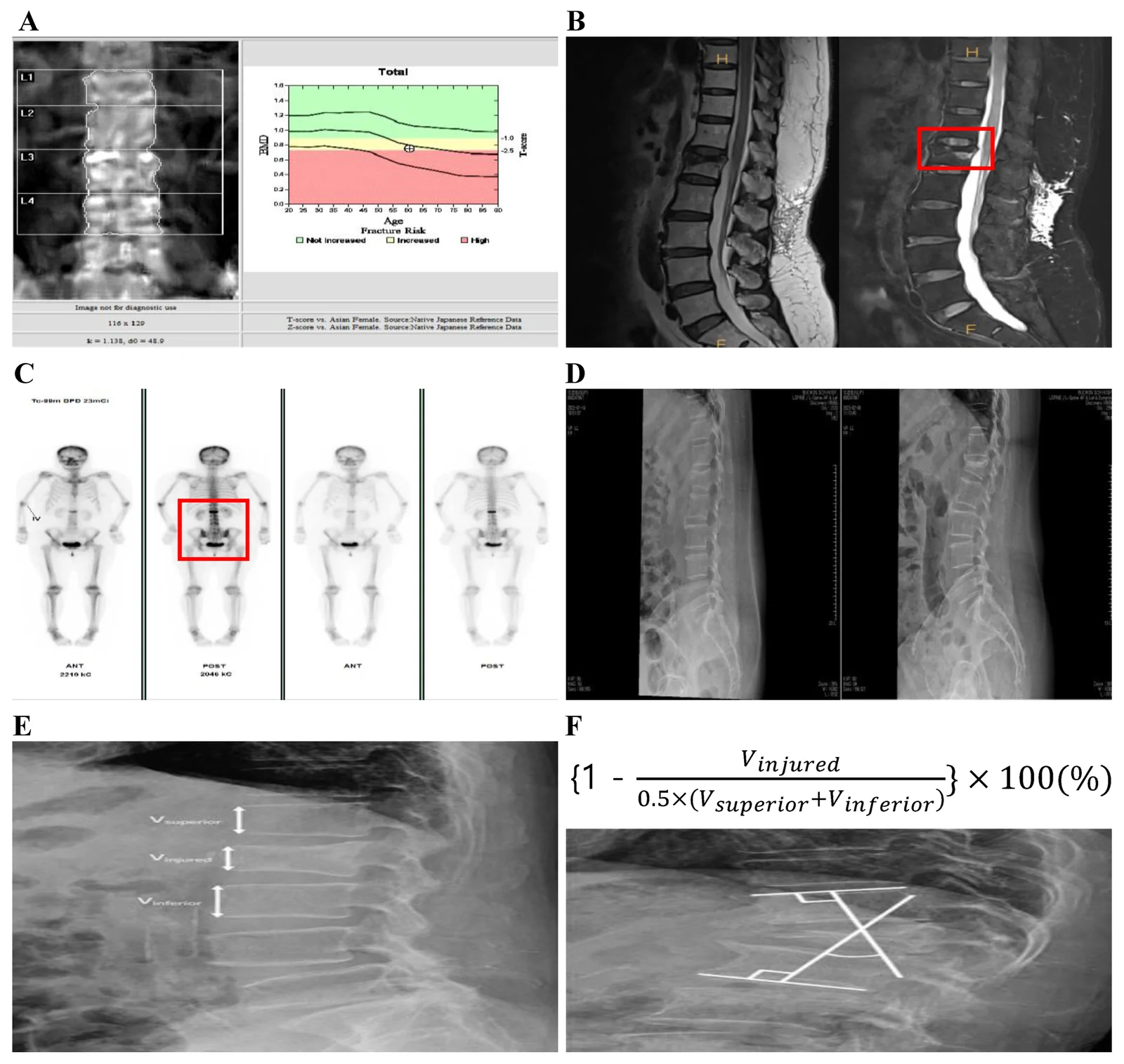
Radiologic assessment of vertebral compression and morphometric parameters in osteoporotic vertebral compression fracture. Radiologic evaluation was performed to assess vertebral compression and quantify fracture severity using standardized morphometric parameters in patients with OVCF. (**A**) DXA analysis demonstrates reduced bone mineral density (BMD), confirming the osteoporotic background associated with fracture susceptibility. (**B**) MRI, (**C**) bone scintigraphy, and (**D**) lateral spine X-ray collectively demonstrate a vertebral compression fracture, including signal changes in the affected vertebra, increased radiotracer uptake indicating active bone remodeling, and structural deformity with vertebral height loss. (**E**) Measurement of anterior vertebral body heights (V_superior, V_injured, V_inferior) and (**F**) quantitative assessment using compression ratio {1 − V_injured/[0.5 × (V_superior + V_inferior)]} × 100 and wedge angle were performed to evaluate fracture severity; the mean wedge angle and compression ratio were 11.35° and 26.11%, respectively.

**Figure 2:**
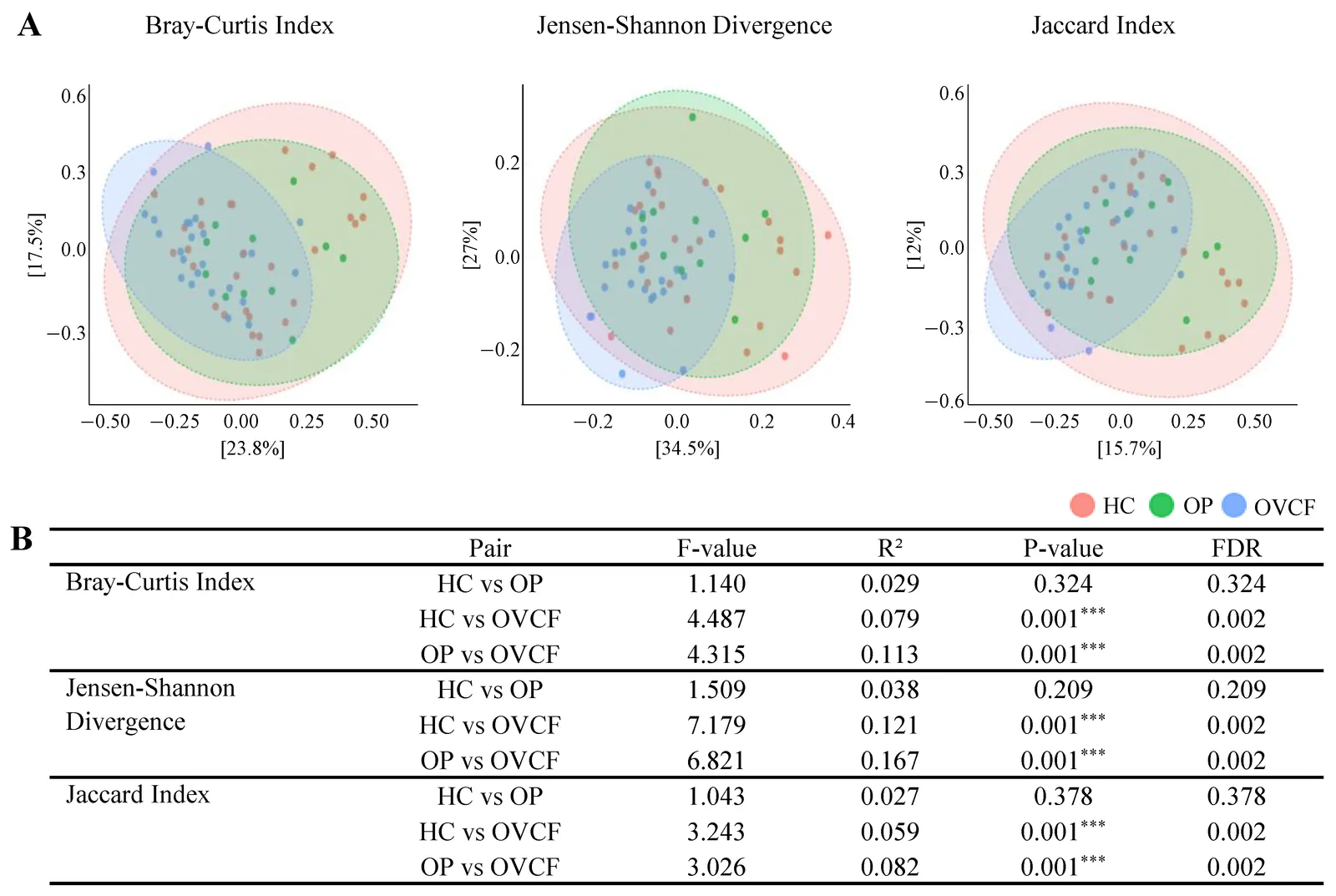
Beta diversity-based differences in gut microbial community structure. Beta diversity analysis was performed to assess differences in gut microbial community structure among the three groups (healthy controls, HC; osteoporosis patients, OP; and osteoporotic vertebral compression fracture patients, OVCF) using PCoA based on the Bray–Curtis index, Jensen–Shannon divergence, and Jaccard index. (**A**) PCoA revealed that the OVCF group was clearly separated from the HC and OP groups across all distance metrics, whereas the HC and OP groups largely overlapped in ordination space. (**B**) Pairwise comparisons showed that the OVCF group was consistently distinct from both the HC and OP groups. At the same time, no clear separation was observed between HC and OP, indicating relatively similar microbial compositions. Statistical analysis was performed using PERMANOVA, and *p*-values were adjusted using the false discovery rate (FDR) method. *** indicates a statistically significant difference between groups (*p* ≤ 0.001).

**Figure 4:**
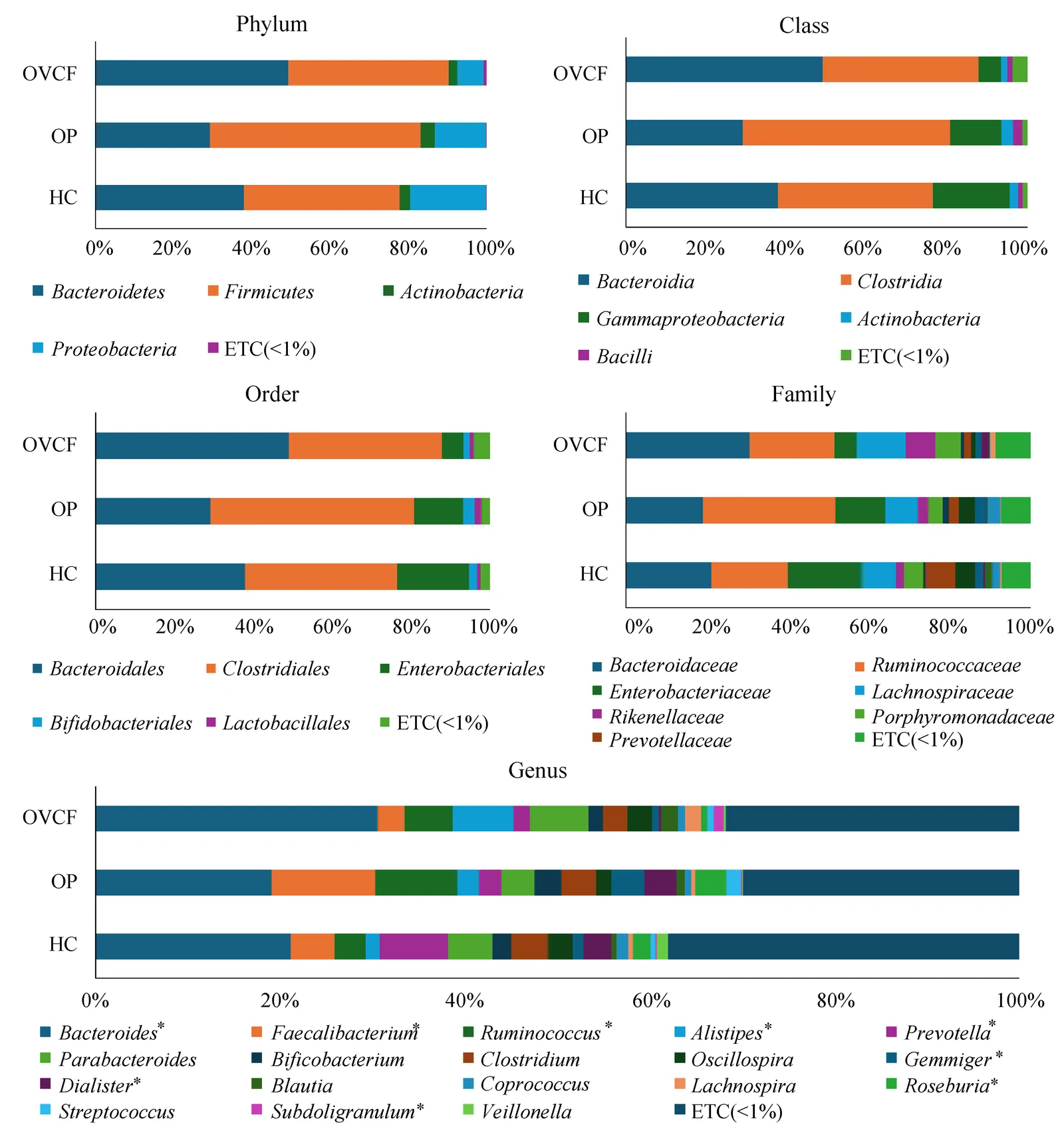
Changes in taxonomic composition of the gut microbiota. Relative abundances of gut microbial communities were compared across phylum, class, order, family, and genus levels in healthy controls (HC), osteoporosis (OP), and osteoporotic vertebral compression fracture (OVCF) groups. No overall significant compositional changes were observed at the phylum–family levels, whereas selective differences were identified at the genus level. Compared to healthy controls, Bacteroides and *Alistipes* were increased in the OVCF group, while *Faecalibacterium*, *Prevotella*, *Subdoligranulum*, *Gemmiger*, *Ruminococcus*, and *Roseburia* showed decreasing trends. Statistical analysis was performed using group-wise comparisons of relative abundance. *p*-values were adjusted using the Benjamini–Hochberg false discovery rate (FDR) method, and asterisks (*) indicate pairwise comparisons that remained statistically significant after FDR correction. Stacked bar plots show the mean relative abundance for each group.

**Figure 5:**
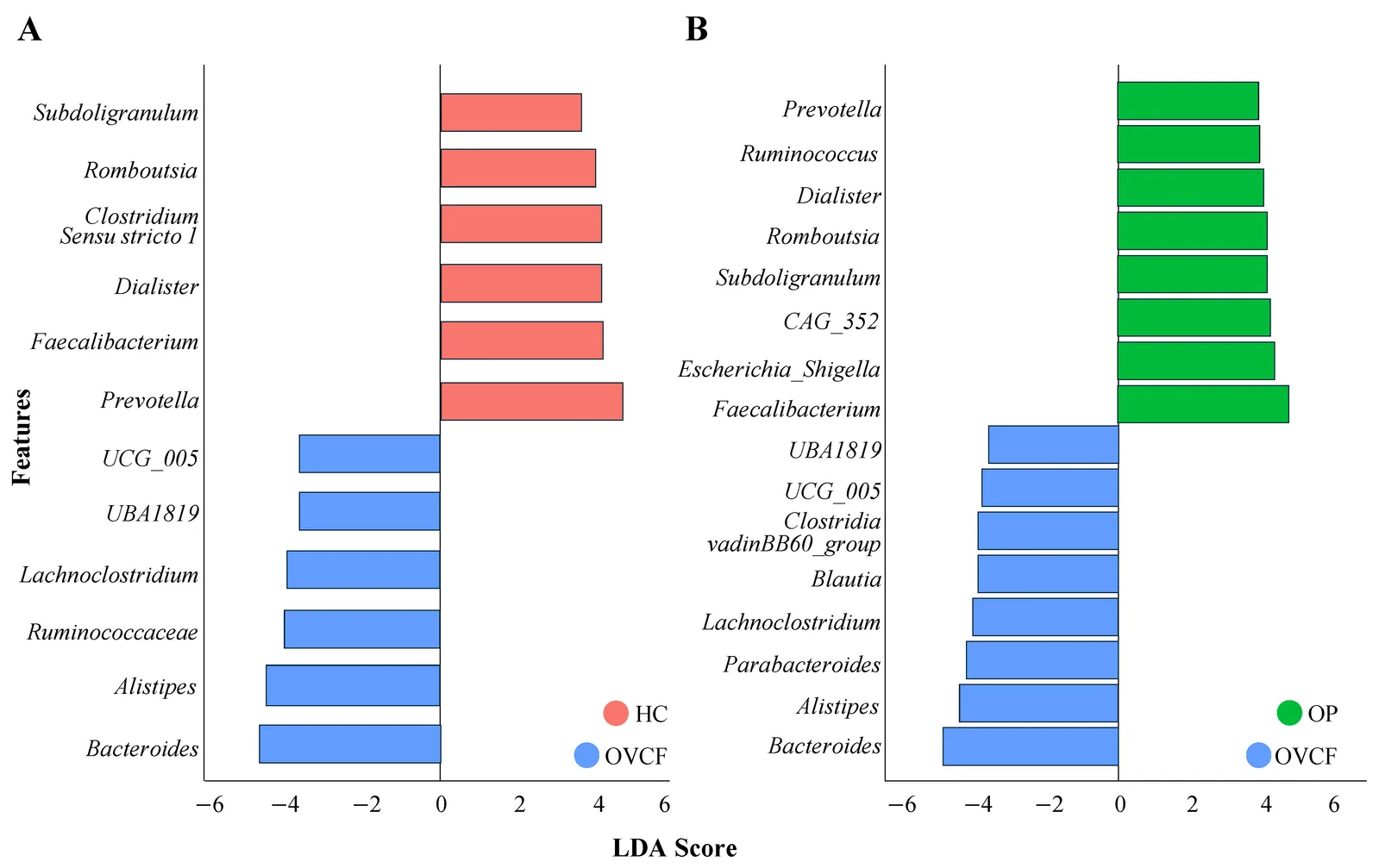
Differentially abundant microbial taxa identified by LEfSe analysis. LEfSe (linear discriminant analysis effect size) analysis was performed to identify microbial taxa that differed significantly between groups based on LDA scores. At an LDA score threshold of ≥4, (**A**) in the comparison between healthy controls (HC) and osteoporotic vertebral compression fracture patients (OVCF), *Prevotella* and *Faecalibacterium* were enriched in HC, whereas *Bacteroides* and *Alistipes* were enriched in OVCF. (**B**) In the comparison between osteoporosis patients (OP) and OVCF, *Faecalibacterium* was enriched in OP, whereas Bacteroides and *Alistipes* were enriched in OVCF. Statistical analysis was performed using LEfSe based on linear discriminant analysis (LDA), where positive LDA scores indicate taxa enriched in the reference groups (HC or OP), and negative scores indicate taxa enriched in the comparison group (OVCF).

**Figure 6:**
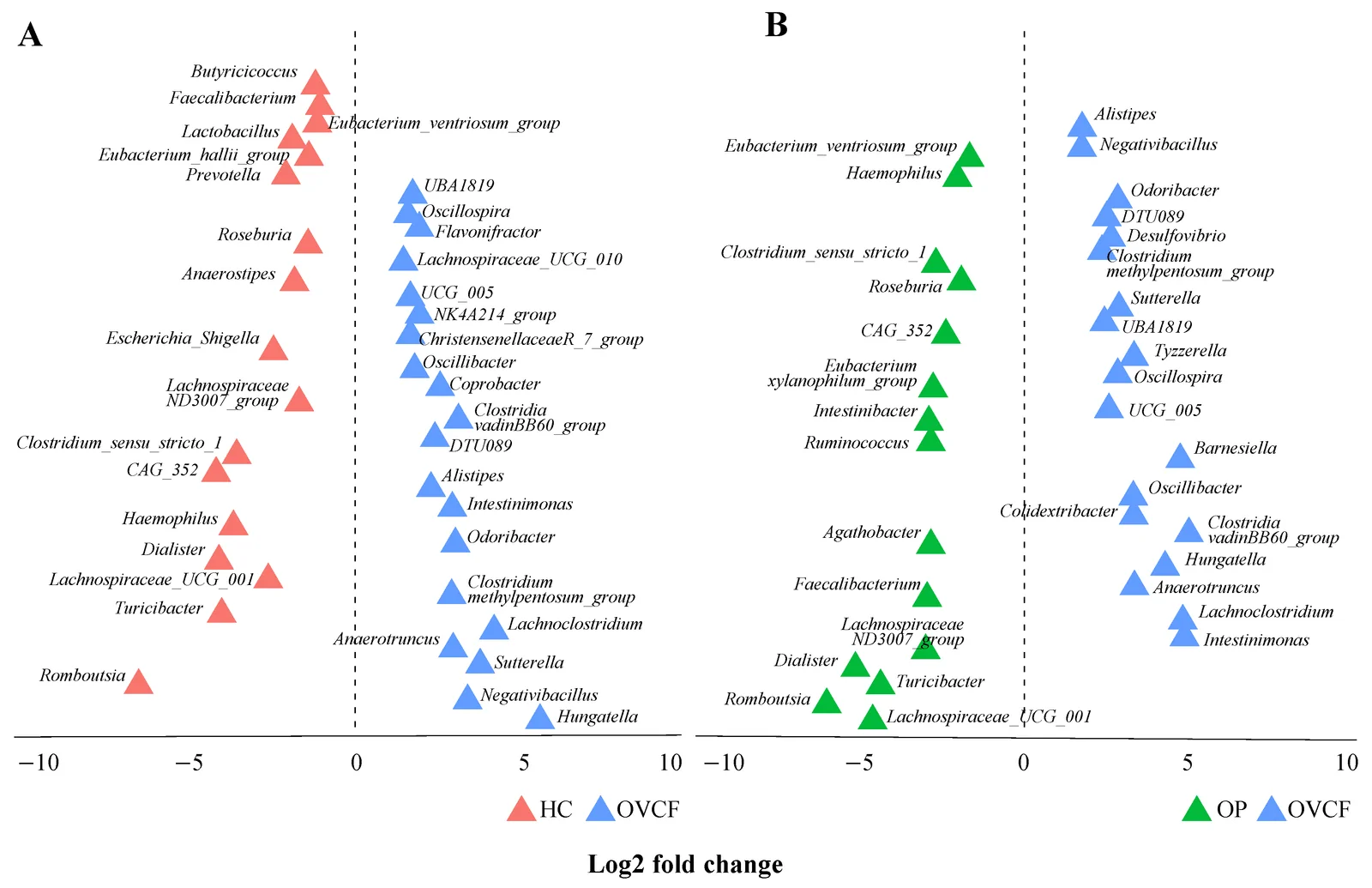
Differential abundance analysis of gut microbial taxa using edgeR. Differential abundance analysis was performed using edgeR to identify microbial taxa associated with healthy controls (HC), osteoporosis (OP), and osteoporotic vertebral compression fracture (OVCF), and to statistically validate biomarkers identified by LEfSe analysis. (**A**) In the comparison between HC and OVCF, multiple taxa showed significant differences. Among these, taxa overlapping with LEfSe-derived biomarkers were highlighted: Prevotella and Dialister were enriched in HC, whereas Alistipes was enriched in OVCF. (**B**) In the comparison between OP and OVCF, Dialister and Alistipes were identified as taxa overlapping with LEfSe-derived biomarkers, enriched in OP and OVCF, respectively. The plots show the log2 fold changes for individual taxa, with each point representing a taxon that remained statistically significant after Benjamini–Hochberg false discovery rate (FDR) correction.

**Figure 7:**
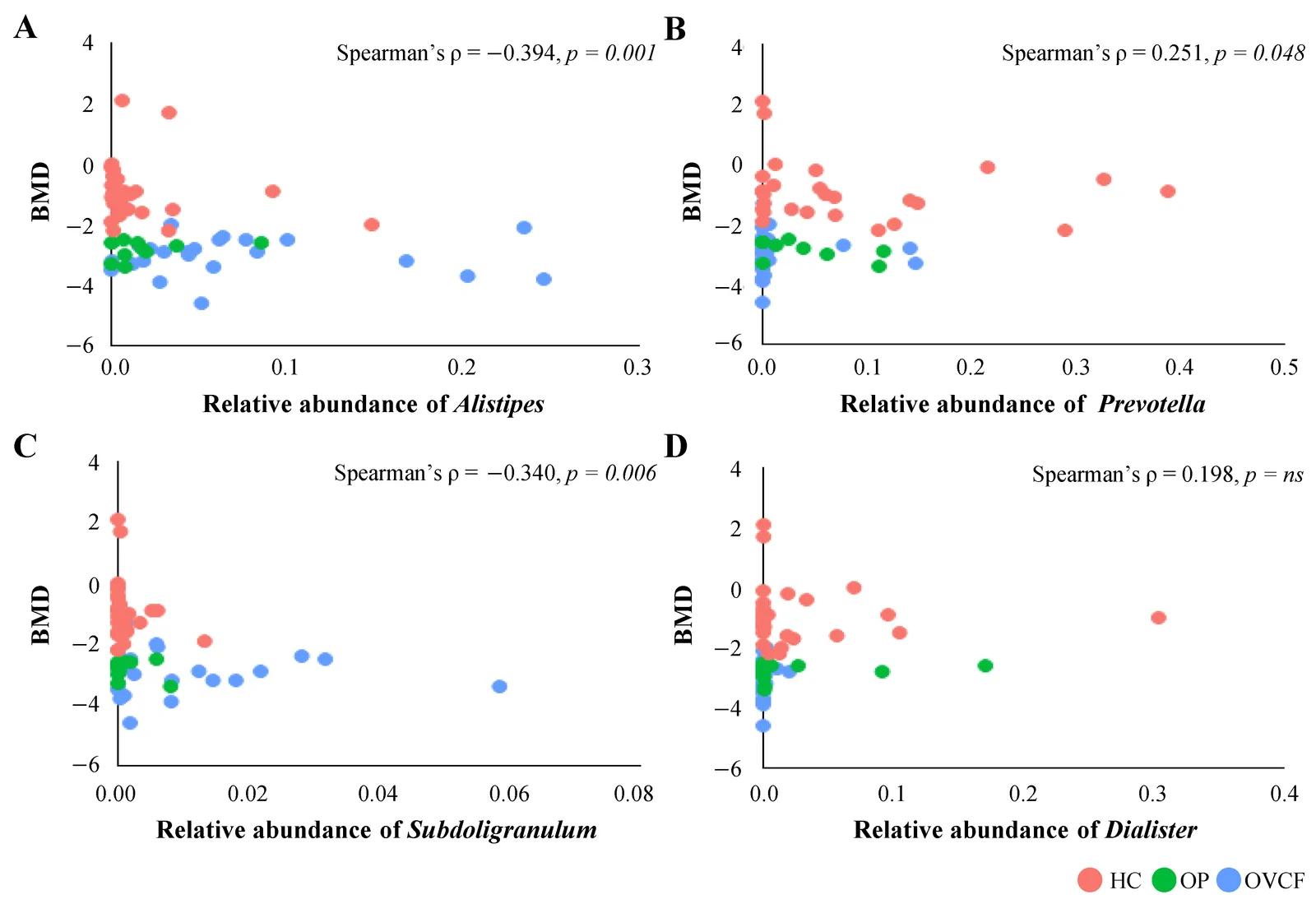
Correlation between bone mineral density (BMD) and relative abundance of specific gut microbial taxa. Spearman correlation analysis was performed to evaluate the relationship between BMD and the relative abundance of specific microbial taxa across the study groups. The analysis focused on taxa identified as biomarkers. (**A**) *Alistipes* showed a negative correlation with BMD, whereas (**B**) *Prevotella* exhibited a positive correlation, both of which were consistent with the previously identified OVCF- and HC-associated biomarkers. (**C**) *Subdoligranulum* showed a negative correlation with BMD but was not consistent with the direction observed in the biomarker analysis, and (**D**) *Dialister* did not show a significant correlation; therefore, neither taxon supported the biomarker-associated trends. Each point represents an individual sample derived from the HC, OP, or OVCF groups. Spearman correlation coefficients (ρ) and corresponding *p*-values are indicated in each panel (*p* < 0.05).

**Figure 8:**
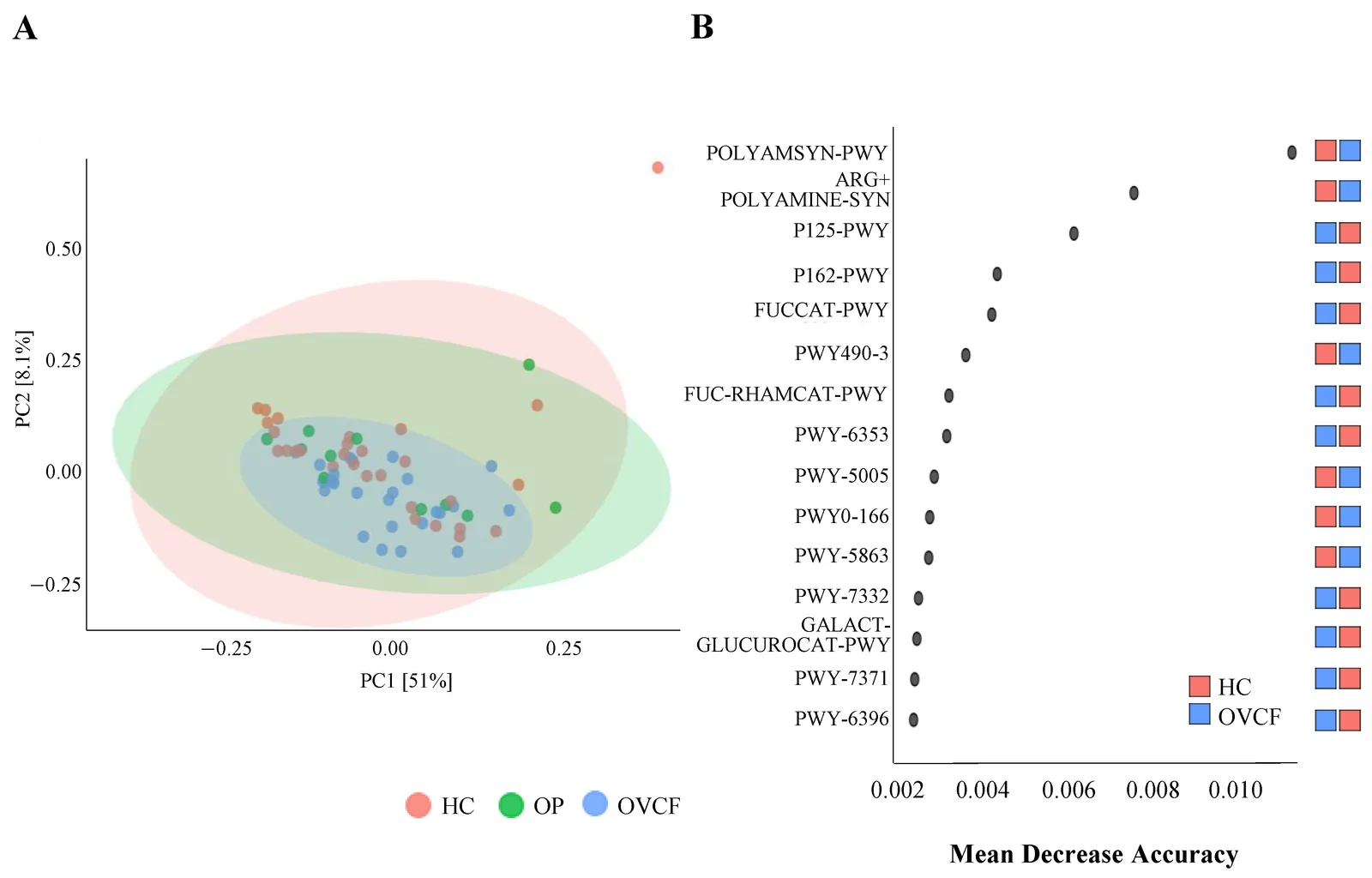
Functional profiling of gut microbial pathways based on MetaCyc analysis and random forest classification. Functional metabolic profiles of the gut microbiome were inferred using MetaCyc pathway analysis, and principal component analysis (PCA) and random forest modeling were performed to evaluate inter-group functional differences and identify key discriminative pathways. (**A**) PCA based on predicted functional pathway profiles showed that HC and OP partially overlapped. In contrast, OVCF exhibited a distinct distribution, indicating functional shifts in microbial metabolic potential associated with disease progression. Each point represents an individual sample, and ellipses indicate the 95% confidence intervals for each group. (**B**) Random forest analysis identified key functional pathways that discriminate HC from OVCF. Notably, pathways related to polyamine biosynthesis and carbohydrate/glucuronate metabolism showed high discriminatory importance in the random forest model, as indicated by the mean decrease in accuracy. These findings suggest that the predicted functional profiles may contribute to distinguishing OVCF from HC; however, they represent inferred functional potential based on 16S rRNA gene sequencing rather than direct measurements of microbial metabolic activity.

**Figure 9:**
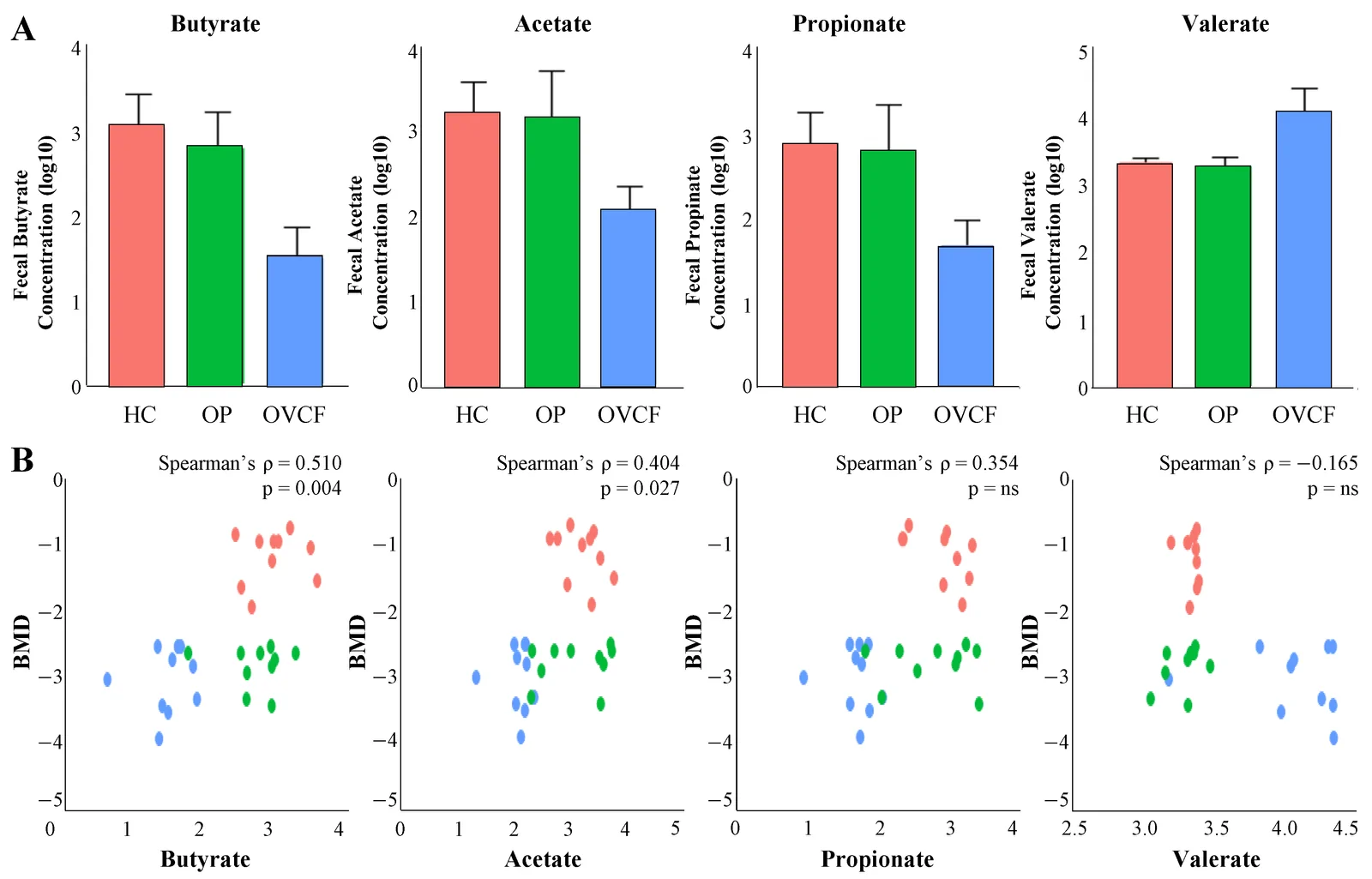
Fecal short-chain fatty acid levels and their association with bone mineral density across study groups. Fecal short-chain fatty acid (SCFA) concentrations were quantified to compare metabolic differences among healthy controls (HC), osteoporosis patients (OP), and osteoporotic vertebral compression fracture patients (OVCF), and Spearman correlation analysis was performed to evaluate their association with bone mineral density (BMD). (**A**) The concentrations of acetate, butyrate, propionate, and valerate were compared among HC, OP, and OVCF groups, showing reduced levels of acetate, butyrate, and propionate in the OVCF group. In contrast, valerate did not show a significant difference. Data were log10-transformed and presented as mean ± standard error. For comparisons among the three groups, the Kruskal–Wallis test followed by Dunn’s multiple comparison test was used. For pairwise comparisons, the Mann–Whitney U test was applied where appropriate. *p*-values were adjusted using the false discovery rate (FDR) method when multiple comparisons were performed. (**B**) Spearman correlation analysis revealed that butyrate, acetate, and propionate showed positive correlations with BMD, whereas valerate did not. Each point represents an individual sample from the HC, OP, or OVCF groups, and Spearman’s correlation coefficients (ρ) and corresponding *p*-values are indicated in each panel. SCFA quantification was performed using 10 fecal samples selected at random from each study group. Data are presented as mean ± SEM.

**Figure 10:**
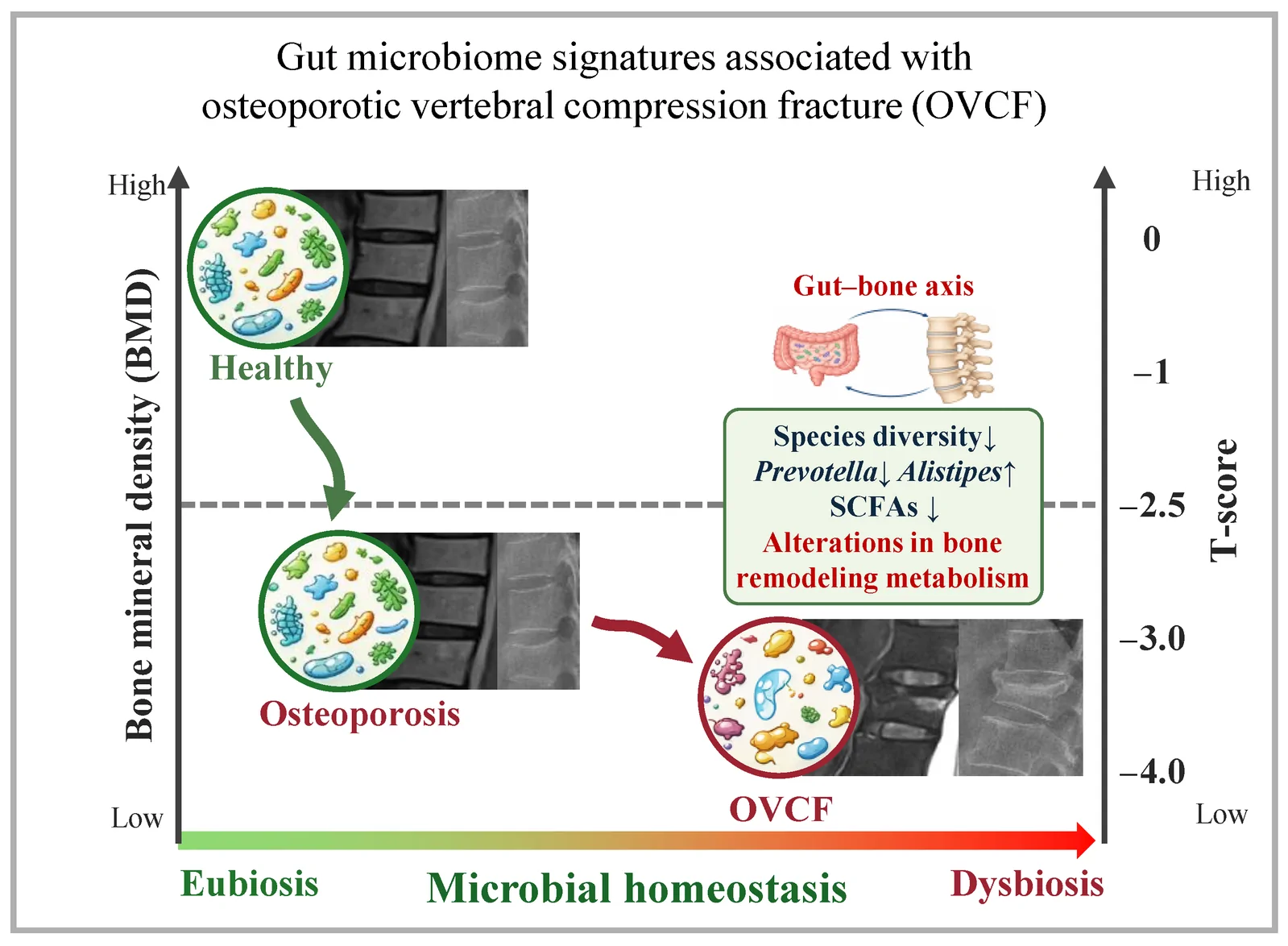
Summary of gut microbiome alterations associated with osteoporotic vertebral compression fracture (OVCF). Osteoporosis (OP) is diagnosed based on decreased bone mineral density (BMD), leading to a reduction in T-score. In more advanced stages, patients may develop osteoporotic vertebral compression fractures (OVCFs), in which fracture occurrence cannot be fully explained by BMD alone and requires a systemic understanding of bone remodeling. The gut microbiome plays an important role in systemic regulation, and the concept of the gut–bone axis has recently been proposed. In this study, changes in the gut microbiome were identified in patients with compression fractures. Microbial diversity was reduced, along with decreased *Prevotella* and increased *Alistipes*. Functional pathway analysis showed increased mucin-derived carbohydrate degradation and decreased polyamine metabolism. These changes were associated with reduced levels of short-chain fatty acids (SCFAs). This may contribute to the development of diagnostic and therapeutic strategies.

## Data Availability

The datasets generated and/or analyzed during the current study are available from the corresponding author upon reasonable request. The clinical and radiological data used in this study were obtained from the Biobank of Soonchunhyang University Bucheon Hospital, a member of the Korea Biobank Network, under approval number 2024ER050100.

## Supplementary Materials

The following supporting information can be downloaded at https://www.mdpi.com/article/10.3390/ijms27198468/s1.

## Abbreviations

HC	Healthy Control
OP	Osteoporosis
OVCF	Osteoporotic vertebral compression fracture
SCFA	Short-chain fatty acids
BMD	Bone mineral density
DXA	Dual-energy X-ray absorptiometry
LEfSe	Linear discriminant analysis effect size
LDA	Linear discriminant analysis
PCoA	Principal coordinate analysis
