# Supplementary material for: Gut Microbiome Signatures Associated with Osteoporotic Vertebral Compression Fracture
Source: Int J Mol Sci. 2026 Sep 23;27(19):8468. doi: 10.3390/ijms27198468 (PMC13665328; doi:10.3390/ijms27198468)
Supplement: Supplementary file 1 [file ijms-27-08468-s001.zip › ijms-4486801-supplementary.pdf]

Supplementary Materials

Gut Microbiome Signatures Associated with Osteoporotic Vertebral Compression Fracture

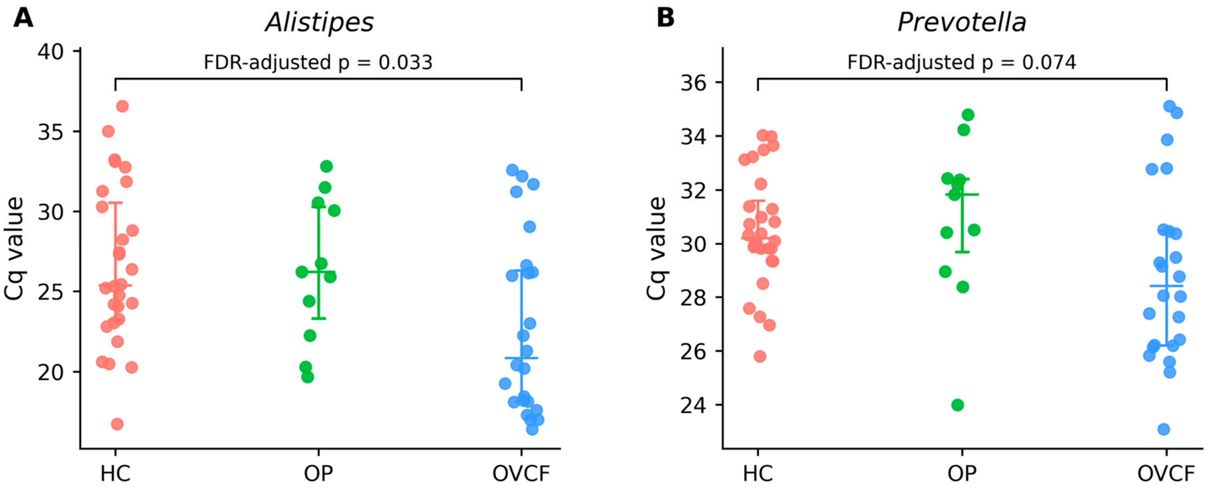

Figure S1. Target-specific qPCR analysis of *Alistipes* and *Prevotella*.

Cq values for (A) *Alistipes* and (B) *Prevotella* were compared among healthy controls (HC), osteoporosis patients (OP), and osteoporotic vertebral compression fracture patients (OVCF). Each point represents the mean Cq value obtained from technical replicate measurements for an individual sample. Lower Cq values indicate greater detection of the target bacterial DNA. Group differences were evaluated using the Kruskal-Wallis test followed by pairwise Mann-Whitney U tests with Benjamini-Hochberg false discovery rate (FDR) correction. *Alistipes* showed significantly lower Cq values in OVCF than in HC (FDR-adjusted p = 0.033), whereas the HC-OVCF comparison for *Prevotella* did not remain significant after FDR correction (FDR-adjusted p = 0.074).

Note: The qPCR analysis was based on target-specific Cq values without normalization to a universal bacterial reference target; therefore, the results are interpreted as target-detection comparisons rather than normalized relative abundance measurements.
